# AutoImpute: Autoencoder based imputation of single-cell RNA-seq data

**DOI:** 10.1038/s41598-018-34688-x

**Published:** 2018-11-05

**Authors:** Divyanshu Talwar, Aanchal Mongia, Debarka Sengupta, Angshul Majumdar

**Affiliations:** 1 Department of Computer Science and Engineering, Indraprastha Institute of Information Technology, Delhi, India; 2 Department of Electronics and Communications Engineering, Indraprastha Institute of Information Technology, Delhi, India; 3 Center for Computational Biology, Indraprastha Institute of Information Technology, Delhi, India

## Abstract

The emergence of single-cell RNA sequencing (scRNA-seq) technologies has enabled us to measure the expression levels of thousands of genes at single-cell resolution. However, insufficient quantities of starting RNA in the individual cells cause significant dropout events, introducing a large number of zero counts in the expression matrix. To circumvent this, we developed an autoencoder-based sparse gene expression matrix imputation method. AutoImpute, which learns the inherent distribution of the input scRNA-seq data and imputes the missing values accordingly with minimal modification to the biologically silent genes. When tested on real scRNA-seq datasets, AutoImpute performed competitively wrt., the existing single-cell imputation methods, on the grounds of expression recovery from subsampled data, cell-clustering accuracy, variance stabilization and cell-type separability.

## Introduction

Bulk RNA sequencing has traditionally been used for parallel screening of thousands of genes in a tissue specimen. Single-cell RNA sequencing (scRNA-seq), on the contrary, enables the measurement of gene expressions in individual cells. Over the past few years, scRNA-seq has revolutionized the field of genomics by facilitating the characterization of phenotypic diversity among seemingly similar cells^1–4^.

Many single-cell transcriptomic technologies have emerged over the last few years, each having its unique capabilities and limitations in terms of throughput, sensitivity, accuracy, and precision. There are two major protocols into which they can broadly be divided: (1) ones that reverse transcribe and amplify full-length mRNA transcripts from single-cell samples in order to extract full sequence information, and (2) ones that amplify only the 5′ or 3′ ends of each transcript, with the aim of counting mRNA molecules for measuring gene expression. Unlike methods that are designed to target specific transcripts (such as microfluidic single-cell qPCR), single-cell transcriptomics suffers from low reverse transcription efficiency (which limits the ability to detect lowly expressed genes) and high levels of technical noise (owing to the paucity of starting RNA)^5^. Hence, only a small fraction of transcriptomes of each cell is captured during the amplification process^6^, so, the gene expression matrix presents significant missing values or dropouts^6–9^. These dropout events introduce technical variability and high noise, making it difficult to analyze the single-cell RNA-seq data^10^. Also, it is quite challenging to distinguish between dropouts and the events of biological gene silencing. As a result, the state-of-the-art nuisance factor rescaling based normalization techniques often fail to denoise the data.

The measured gene expression matrix can be perceived as a partially observed version of the complete gene expression matrix (with no dropout events). The problem is akin to that of collaborative filtering; therein the ratings matrix is partially observed and the goal is to estimate the complete matrix given the partially observed ones. Our problem is to recover the complete gene expression matrix by imputing the dropouts. Traditional strategies to solve the matrix completion problem include (1) nuclear norm minimization, (2) matrix factorization and (3) autoencoders. Autoencoder based methods generalize better and are less prone to overfitting for a data restricted problem like ours, as the number of parameters that are to be learned/estimated is much smaller than the number of learnable parameters in matrix factorization or nuclear norm minimization (more on this in the methods section).

**Figure 1:**
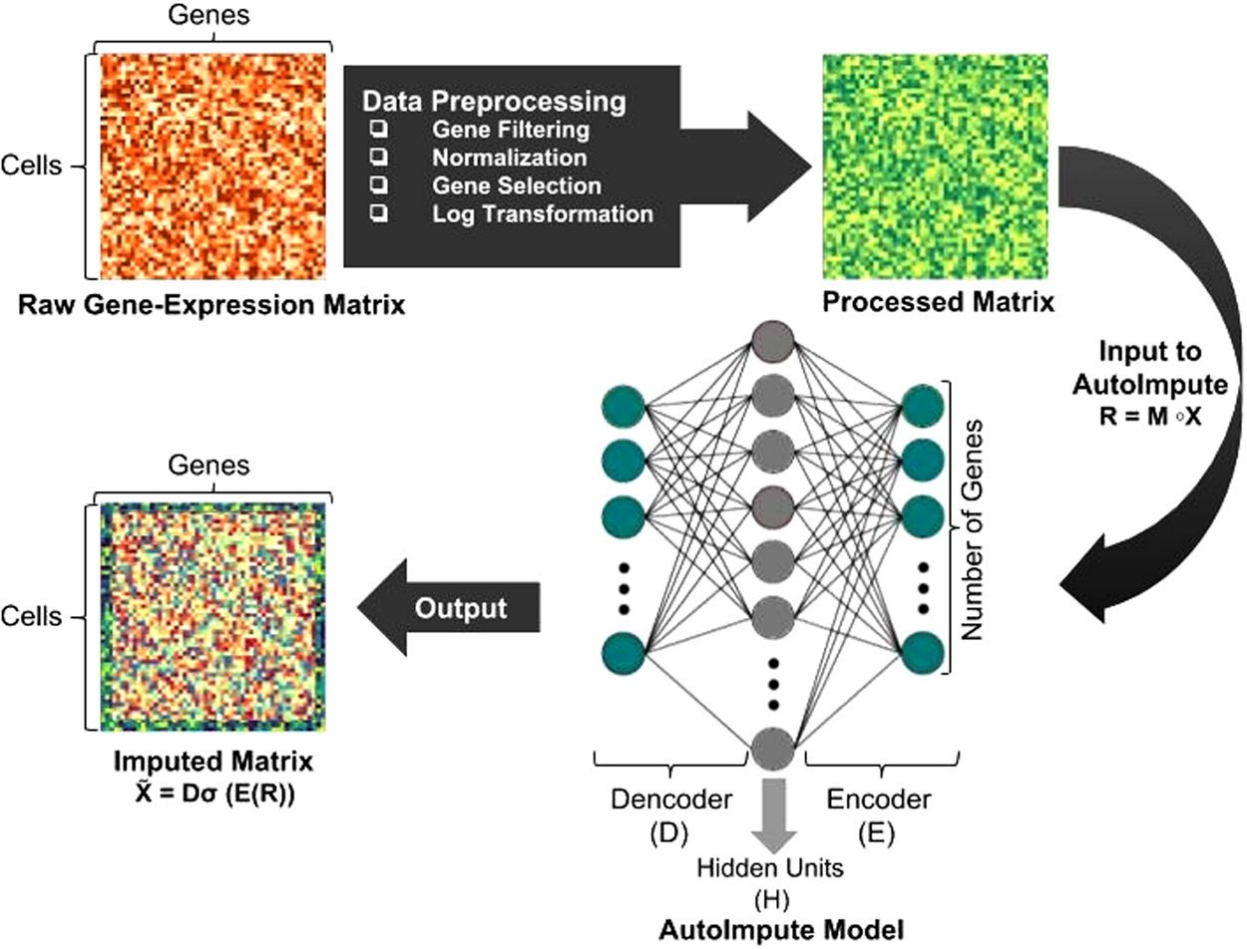
AutoImpute pipeline: The raw gene expression data is filtered for bad genes, normalized by library size, pruned by gene-selection and log transformed. Then, the processed matrix is fed to the AutoImpute model for learning expression data representation and finally reconstructing the imputed matrix.

In this work, we propose AutoImpute (Fig. 1), a novel method for sparse gene expression matrix imputation using overcomplete autoencoders. AutoImpute learns the inherent distribution of the input scRNA-seq data and imputes the missing values accordingly with minimal modification to the biologically silent gene expression values.

We compare the performance of AutoImpute with the existing imputation methods on nine independent datasets. Our proposed method performs competitively as evaluated on the grounds of expression recovery from subsampled data, cell-clustering accuracy, variance stabilization across cells of identical type and cell-type separability. Also, AutoImpute is the only method which is capable of performing imputation on the largest of the nine datasets in question, PBMC (having ~68,000 cells), without exhausting memory.

## Related Work

Recently, attempts have been made to devise imputation methods for single-cell RNA sequencing data, most notable among these are MAGIC, scImpute, and drImpute^11–13^. MAGIC uses a neighborhood-based Markov-affinity matrix and shares the weight information across cells to generate an imputed count matrix.

On the other hand, for a zero expression value, scImpute first estimates the probability of it being a dropout. It uses a Gamma-Normal mixture model to take into account the dropout events. Zero expressions, which are likely to be dropouts are then estimated by borrowing information from similar cells. scImpute has been shown to be superior as compared to MAGIC. Another method, drImpute, repeatedly identifies similar cells based on clustering and performs imputation multiple times by averaging the expression values from similar cells.

Our approach, AutoImpute is motivated by a similar problem^14^ of sparse matrix imputation frequently encountered in recommender systems a.k.a collaborative filtering in information retrieval. The problem is well illustrated with the following example. When designing a recommender system for movies (like in Netflix), we are given a user-movie rating matrix in which each entry (*i*, *j*) represents the rating of movie *j* by user *i* only if the user *i* has watched movie *j* and is otherwise missing. The problem now is to predict the remaining entries of the user-movie matrix, to make suitable movie recommendations to the users.

With the aim to impute the sparse user-movie rating matrix in the aforementioned problem, various algorithms have been proposed; the most popular ones amongst which are Matrix Factorization^15,16^ and Neighborhood Models^17^. The use of latent factor models like those based on the autoencoders^18^ have been rising, stemming from the recent successes of (deep) neural network models for vision and speech tasks. Justifying their popularity in the recent years autoencoder based matrix imputation methods outperform the current state-of-the-art methods. So, we adopt and deploy this idea to address the problem of dropouts in scRNA-seq data.

## Results

### Handling Dropouts and Retaining True Zeros

Due to the lack of the starting RNA material single-cell RNA-seq experiments suffer from high dropout rates. As a result, expression datasets often contain an excessive number of zero expression values compared to typical bulk expression datasets. While a majority of these zero expression values manifest technical dropouts, a sizeable fraction of these is caused by biological silencing of the concerned genes. There is no confirmatory method to zero in on the source of origin of the zero expression values. To circumvent this, we made use of matched bulk expression profiles. Bulk RNA-seq experiments involve millions of cells, thus do not suffer from dropouts. To this end, we considered zero expressions in a homogeneous bulk cell populations derived from myoblast cell line (Trapnell data^19^) to be indicative of true biological silencing. We imputed the matched single cell expression data from the same cell line using various imputation methods. We conjectured that a reasonable imputation technique should retain zero expressions due to biological silencing, whereas impute the dropout cases.

**Figure 2:**
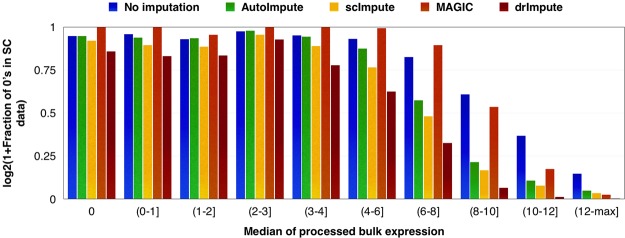
Handling dropouts and retaining “true zeros” - AutoImpute, scImpute, and MAGIC successfully recover the majority of true zeros in the data and don’t confuse them with dropouts (which need imputation). As the gene expression in bulk-RNA seq data enriches, the zeros in unimputed data (which essentially are dropouts) are filled in with counts in imputed data from AutoImpute, scImpute and drImpute, showing a drop in the fraction of zeros; while Magic mostly shows a high fraction of near-zero imputed values.

The existing techniques were tested on the aforementioned experiment, by first processing the bulk RNA-seq data using TMM normalization, followed by log-transformation; to check for composition bias. Then, the median of gene expression across three different biological replicates taken from cells of undifferentiated myoblasts was considered to create expression bins. The genes with zero expression in the bulk data were considered to be biologically silent and are assigned a separate bin. Rest of the expressed genes were grouped based on their median expression. For each set of genes belonging to a bin (found using the bulk data), the fraction of zeros (*number of zeros in the set* ÷ *total count of the set*) in the imputed single-cell expression data is reported on a natural logarithmic scale in the Fig. 2.

Among the existing methods, AutoImpute is the most conservative wrt. imputation of the biologically silent genes, i.e., it behaves ideally in retaining the most amount of true zeros present in the data, certainly better when compared with scImpute and drImpute. As we move right along the axis with the median expression bins in Fig. 2, we notice a drop in the fraction of imputed zeros for AutoImpute, scImpute, and drImpute. This trend (similar for all three methods) is indicative of the effectiveness of these methods in imputing dropout values. Though the data imputed using MAGIC conserves the biologically silent genes equally well, it fails to recover most of the dropouts present in the un-imputed data. Although AutoImpute recovers less number of missing values for genes which are reasonably expressed, it performs better for other metrics of evaluation.

### Gene expression recovery

Though it hasn't been proven in a systematic manner, dropouts are often modeled as a Poisson process^20^. We, to avoid any bias, simulate dropouts by muting the gene expression randomly. For seven gene expression datasets (Blakeley, Jurkat-293T, Kolodziejczyk, Preimplantation, Quake, Usoskin and Zeisel), we artificially masked (removed) a certain percentage of the total non-zero expression values at random, and used the imputation techniques to estimate the expression values at these missing locations. For this, five incomplete data matrices per dataset were created with the masking of varied portions of data (10 to 50%). As mentioned in the introductory section, we could not carry out these experiments on the PBMC dataset owing to its size (given the limitations in resources and time constraints).

**Figure 3:**
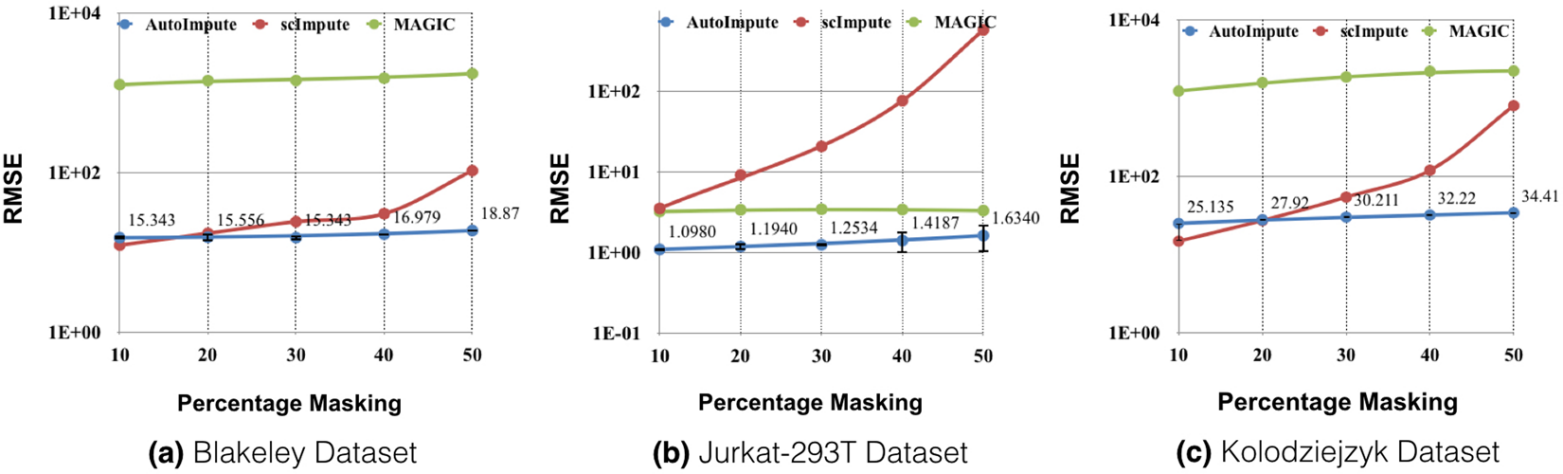
Variation of RMSE between recovered and actual values in scRNA-seq data with increasing masking percentage computed for AutoImpute, scImpute, and Magic. AutoImpute exhibits best expression recovery with lowest RMSE for all the datasets.

To compare among the imputation methods, we use Normalized Mean Squared Error (NMSE), Root Mean Squared Error (RMSE) and Mean Absolute Error (MAE) between the corresponding recovered and full expression dataset as evaluation metrics; these are standard metrics for studying recovery in machine learning. Owing to the random nature of weights initialization in an autoencoder, all the experiments were run 10 times and both mean and variance of the aforementioned metrics are reported **(**Supplementary Table S2**)**. From the results shown in Fig. 3 and Supplementary Table S2, we observe the following:

- The reconstruction error from AutoImpute is in general better than other imputation strategies, RMSE and MAE being always lesser in majority of datasets, while NMSE being less than all methods except MAGIC.
- As the observability of input expression matrices to various imputation strategies increases, ideally any imputation method should have improved performance. Talking of all metrics NMSE, RMSE, and MAE, this trend is only observed for AutoImpute and ScImpute and not always for MAGIC.

The imputations from drImpute algorithm removes some insignificant input genes, normalizes the input and log-transforms the data as part of its preprocessing. Since muting the gene-filtering step in drImpute pipeline was not possible, input and output expression matrices have different dimensions, hence, the error metrics were not calculated for drImpute.

### Improvement in clustering accuracy

A large number of dropouts in single-cell RNA sequencing data can give a false view of expression levels, which might compromise the integration and interpretation of the data. Such kind of technical and biological noise is bound to trick a clustering algorithm which aims to cluster cells of similar types.

This issue should be fixed by an imputation method which correctly recovers single-cell expression data. So, We applied K-means clustering algorithm on the log-transformed datasets to observe how well do the imputation strategies improve the cell clustering results. The performance metric we used to evaluate the correspondence between the original annotations and K-means assigned annotations is Adjusted Rand Index (ARI)^21^.

The initialization parameter *K* (number of clusters) in K-means algorithm has been set to the number of unique cell-types. The results show that our approach gives the best Adjusted Rand Index amongst all the state-of-the-art imputation strategies for five out of the eight datasets we tested on (as shown in Table 1). For other datasets too, the performance was comparable, with ARI always better than the data without any imputation.

**Table 1:** Comparison of different imputation techniques using the Adjusted Rand Index from K-means clustering results.

Datasets	No Imputation	scImpute	drImpute	Magic	AutoImpute
Blakeley	0.483	**0.7713**	**0.7713**	0.3907	**0.7695**
Jurkat-293T	0.9871	0.9859	0.9100	0.9719	**0.9917**
Kolodziejczyk	0.2944	0.5408	**0.6285**	0.1356	0.5667
PBMC	0.2687	—	—	—	**0.2850**
Preimplantation	0.385	0.391	0.3942	0.3715	**0.5356**
Quake	0.5497	0.4451	**0.5936**	0.3224	0.5740
Usoskin	0.3357	0.2868	0.0024	0.0586	**0.4426**
Zeisel	0.4698	0.3340	**0.5224**	0.2722	**0.5213**

Of note, each of the other existing methods exhausted the system memory when applied on the PBMC dataset (hence shown by–entries in Table 1).

### Variance stabilization

We examined the gene expression variance of cells within a cell-type. A reasonable imputation should reduce the intra-subpopulation variation of the gene expression.

We randomly picked up a subpopulation type from all eight datasets and measured the coefficient of variation, CV (a standard measure of relative variability calculated as the ratio of the standard deviation to the mean, also called relative standard deviation) for individual genes across these cells for unimputed data as well as data imputed with various imputation methods.

It was found that AutoImpute stabilized the intra-subpopulation gene expression variance the most in three (Jurkat, Preimplantation, and PBMC) datasets, performs better than all imputation methods except MAGIC in two datasets, and gives a reasonable improvement in CV for others too.

**Figure 4:**
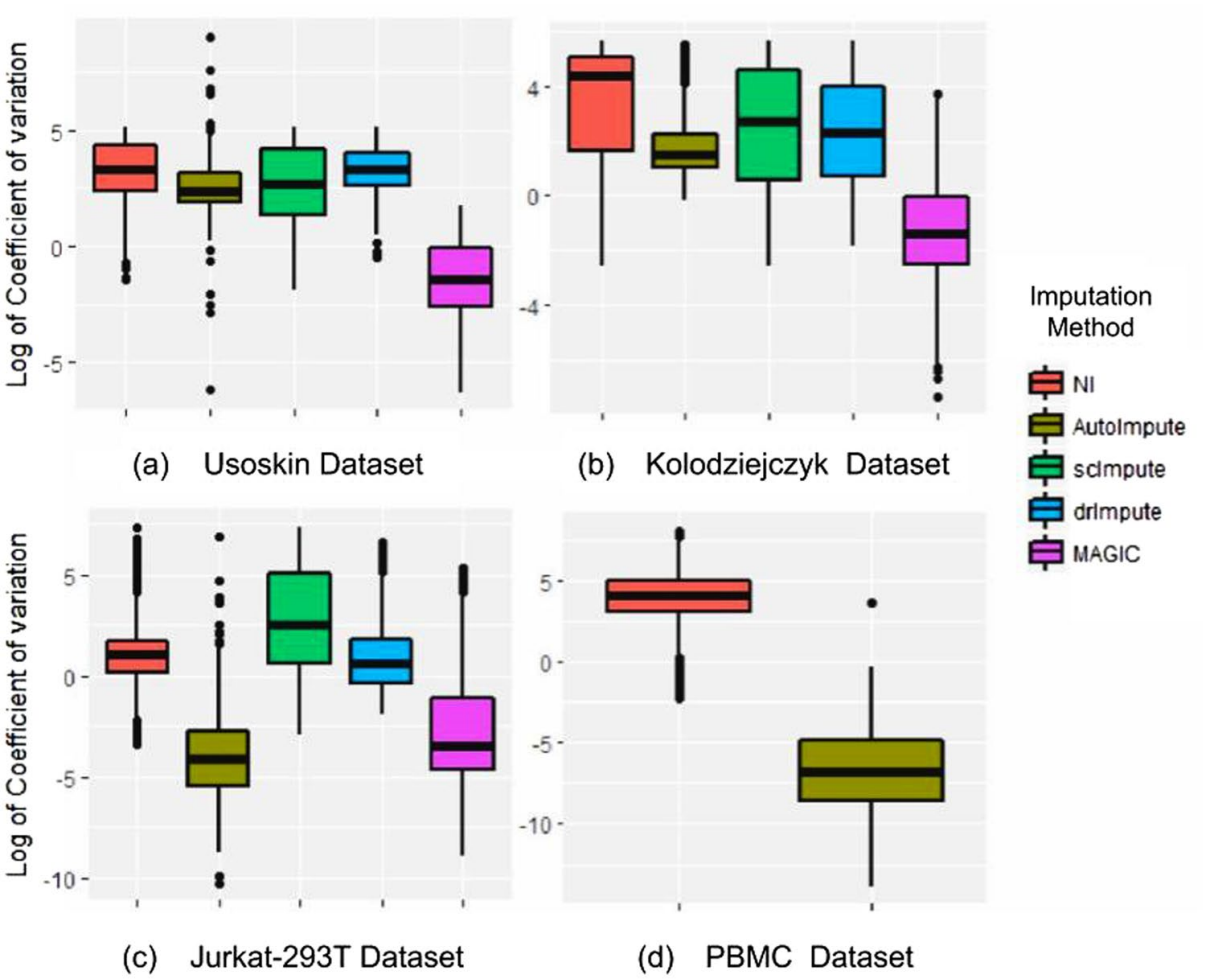
Boxplots comparing log of coefficient of variation computed for individual genes, by various techniques, across cells within random subpopulation taken from (**a**) Usoskin dataset; (**b**) Kolodziejczyk data; (**c**) Jurkat-293T dataset; and (**d**) PBMC dataset. AutoImpute shows significant stability in the intra-subpopulation coefficient of variation.

The plot in Fig. 4 summarizes the natural logarithmic value of the coefficient of variation for genes across the cell subtypes from four datasets in the form of boxplots. Before computing the coefficient of variance, we perform the anti-log operation on the imputed data obtained from the techniques which took log-transformed data as input. We use natural log to amplify the fractional differences between the values and hence make the difference more noticeable in the figure.

### Improvement in cell type separability

Visually well-separated cell-types in single-cell expression data is indicative of more valid biological conclusions from the data. A good imputation strategy should improve the separability of various cell-type subpopulations. To assess this, We reduce the gene expression to two dimensions by applying Principal Component analysis on it, and further plotting the cell transcriptomes in 2D space, coloring each cell by its annotation. Quantitatively, we observe the average of silhouette index values, an unsupervised metric to inspect how well each method groups/separates the cells from various subpopulations.

**Figure 5:**
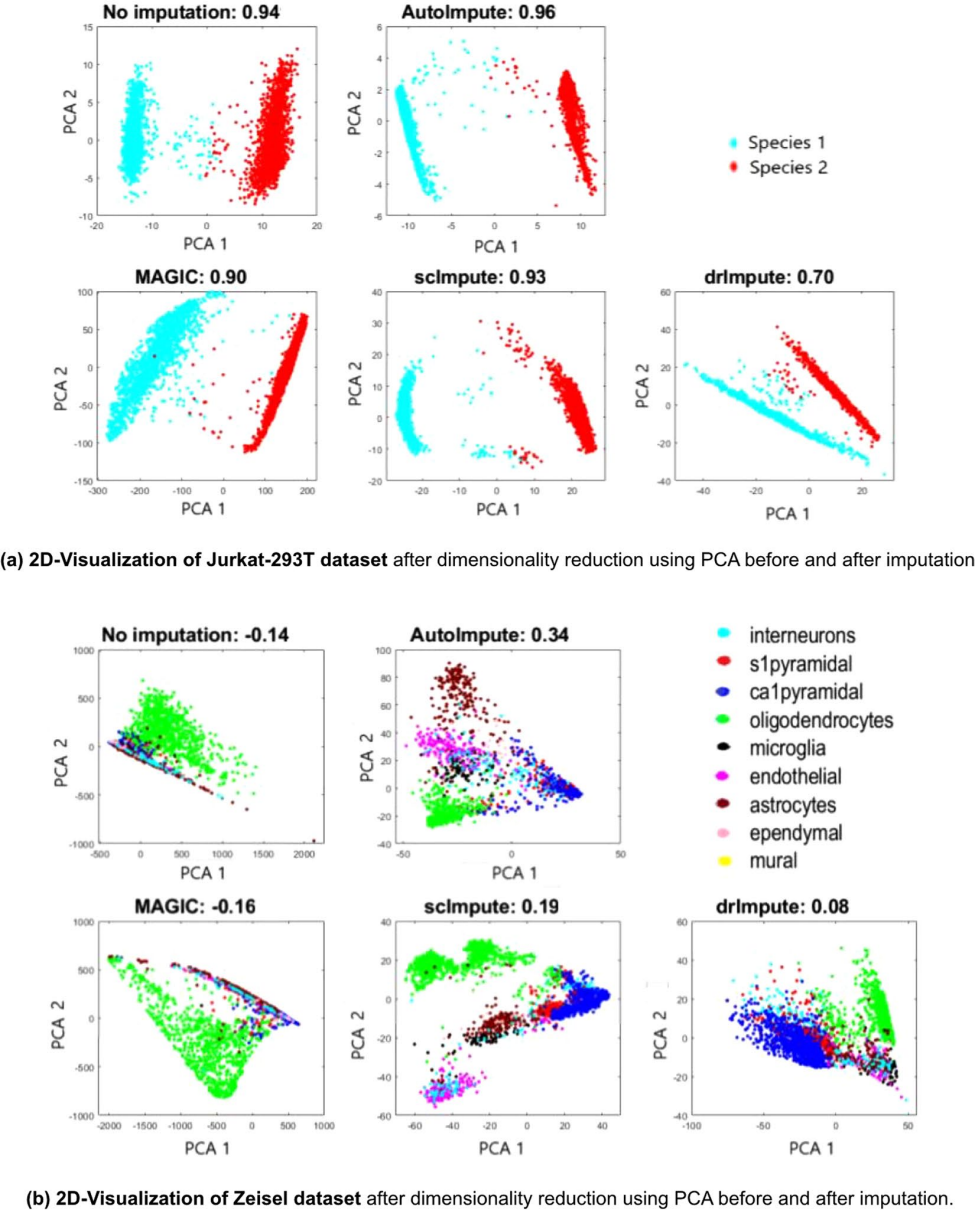
Plots showing 2D-Visualization (after dimensionality reduction using PCA) and average silhouette values for (**a**) Jurkat-293T and (**b**) Zeisel datasets before and after imputation. AutoImpute groups the same cell-types together showing improved separability as depicted.

Figure 5 shows the results on Jurkat and Zeisel datasets. Average silhouette index values for all the datasets have been summarized in Supplementary Table S4.

## Discussion and Conclusion

Motivated by the recent success of neural networks, we present an imputation model which uses an over-complete autoencoder, AutoImpute. It learns the inherent distribution and patterns of gene expression in individual cells to reconstruct an imputed version of the expression matrix by projecting the expression profiles into a high dimensional latent space. AutoImpute, to our knowledge, is the very first scRNA-seq imputation method which bypasses the statistical modeling of single-cell expression by implementing a state-of-the-art machine learning technique. Of note, scImpute, a leading imputation technique for scRNA-seq data models gene expression using parametric distributions. We conjecture that such assumptions may not hold as the noise distribution changes with the introduction of novel platforms for single-cell transcriptomics. A neural network based technique works by minimizing a specific loss function and therefore, in principle, is not sensitive to the marginal distribution of the gene expressions.

The various experiments demonstrated in this article portray the competitive performance of AutoImpute with respect to, the existing techniques. AutoImpute out-performs various state-of-the-art techniques on the grounds of a number of evaluation metrics (discussed), and also scales well to huge datasets with thousands of transcriptomes. Also, in the handling-dropouts experiment, AutoImpute (and scImpute) depict an intuitive trend of the true-zeros recovery along with an effective dropout imputation (with the amplification of gene expression), which implies that the algorithm successfully imputes missing values with minimal disturbance to the biologically silent genes in the recovered matrix.

With the amplification of gene expression in bulk RNA-seq data, scImpute and AutoImpute have been observed to recover a higher number of dropout events as compared to MAGIC. Also, Fig. 5 manifests that MAGIC fails to preserve the structure of single-cell expression data and does not improve or even retain the cell-type separability. Of note, MAGIC stabilizes the variance (Fig. 4) the most across selected cell types for a majority of datasets. Thus, variance stabilization alone might not be a reliable metric for evaluation of scRNA-seq data imputation methods.

Through this work, we hope to bridge the gap between two seemingly disjoint research areas, collaborative filtering and bio-informatics and anticipate to establish a symbiotic relationship between the two.

## Methods

### Matrix Completion Techniques–Literature Review

In scRNA-seq data, only a fraction of transcriptome of each cell is captured, due to insufficient quantities of input RNA. This makes the measured gene expression a partially observed version of the complete data (with no dropout events). We aim to impute these dropouts or missing values by finding this complete version of the gene expression data. The formal model for measurement can be expressed as follows:

1 $$R=M\circ X$$

where $\circ$ is the Hadamard product, M is the binary mask: having 1’s where R contains a non-zero entry and 0’s elsewhere, and X represents the count matrix with no dropouts, which needs to be estimated.

This is an under-determined linear inverse problem and hence has infinitely many solutions, and therefore multiple ways to solve it. The problem is akin to that of recommender systems a.k.a collaborative filtering^15,17,22,23^ in information retrieval. In there, X is the unknown ratings matrix of users and items while R is its partially observed version. The goal is to recover the complete ratings matrix, so that (estimated) highly rated items can be suggested to users.

There are two class of methods to solve (1). The first one linear interpolation approach^15,17,22^, where the missing values are filled by heuristically defined interpolation weights (in the row or column direction). The advantage of this approach is its interpretability, however such naϊve techniques do not yield very good results.

The other approach is more abstract based on latent factor model^15,23^. In the simplest form, it assumes that X is a low rank matrix and hence can be expressed as a product of one thin (*U*) and one fat (*V*) matrix: *X* = *UV*. Incorporating this model into (1) leads to the standard matrix factorization problem^15^.

2 $$R=M\circ (UV)$$

This is usually solved by alternating least squares technique to recover *U* and *V*. Once they are recovered, it is trivial to obtain X.

The factorization approach (2) is bi-linear and hence non-convex, and therefore suffers from non-convergence and non-uniqueness. A mathematically better, albeit more abstract approach to solve (1) is to directly solve for a low-rank *X* from (1). This is achieved via nuclear norm minimization^23^. Such a nuclear norm minimization technique has been used for estimating missing values in bulk data^24^.

One cannot have any insight into heuristic neighborhood based models, but the one can understand the latent factor models by analyzing the information content of (1). Assume that *X* is a matrix of dimensions *m* × *n* but with rank *r*. Then the number of degrees of freedom in X is *r*(*m* + *n* − *r*), which is much smaller than the size of the matrix. Therefore as long as the number of measurements in R is larger than the number of degrees of freedom, one can expect to recover the *X*.

In recent times, with the advent of representation learning, autoencoder based models have been improving the accuracies for collaborative filtering^18,25–27^. An autoencoder is a self-supervised neural network, i.e. the input and the output are the same. Therefore, the autoencoder basically learns to model identity. However, since the number of nodes in the representation/hidden layer is not the same as the input dimensionality, autoencoders learn interesting structures from the data.

Given the success of autoencoders in collaborative filtering, we propose to employ the same model for our problem. Since autoencoders may not be well known to the community, we review them briefly in the next sub-section.

### Autoencoder

An autoencoder consists of an encoder E and a decoder D. The encoder first maps the input data *Y* into the latent space (*H*) -

3 $$H=\varphi (EY)$$

where *ϕ* is the activation function; usually tanh or sigmoid.

The decoder (*D*) projects the latent space (*H*) back to the input space; this is given by -

4 $$X=DH=D\varphi (EX)$$

During the training phase, the encoder and the decoder are learned by minimizing (usually) the Euclidean cost function.

5 $$\mathop{{\rm{\arg }}\,{\rm{\min }}}\limits_{D,E}\parallel X-D\varphi (EX){\parallel }_{F}^{2}$$

There are several variants of the autoencoder model. Stacked autoencoders^28^ are created by nesting one autoencoder inside the other. Mathematically this is represented as,

6 $$\mathop{{\rm{\arg }}\,{\rm{\min }}}\limits_{D^{\prime} s,E^{\prime} s}||X-{D}_{1}\varphi ({D}_{2}\,\mathrm{...}\,\varphi {D}_{N}(\varphi ({E}_{N}\,\mathrm{....}\,\varphi \,{E}_{1}(X{\mathrm{)...)||}}_{F}^{2}$$

Then there are regularized autoencoder. In a generic fashion, they can be expressed as -

7 $$\mathop{{\rm{\arg }}\,{\rm{\min }}}\limits_{D,E}||X-D\varphi (EX{)||}_{F}^{2}+\lambda \Re (E,D)$$

where *λ* is the regularization coefficient, and the regularizer ℜ can range from simple Tikhonov penalties on the encoder and decoder to more complex priors like sparsity^29,30^, rank deficiency^31^ and contractive penalties like the l2-norm of the Jacobian^32^. Traditionally autoencoders have been used for pre-training neural networks^33^. However there are a few studies showing their application in solving inverse problems^34,35^ and dimensionality reduction.

### AutoImpute: The technique

Given the similarity between our problem and collaborative filtering, we leverage the autoencoder based imputation approach from the prior studies (in collaborative filtering) to address the problem at hand. We aim to use overcomplete autoencoders to capture the distribution of the given sparse gene expression data and hence, regenerate a complete version of the same. This is done by feeding the sparse gene expression matrix - *M*$\circ$*X* as input to the autoencoder, and then, training it to learn the encoder and decoder functions which best regenerate the imputed expression matrix *X* (with no dropouts), by back-propagating the errors, only for the non-zero counts present in the sparse matrix *M*$\circ$*X*.

The perfect imputation is achieved by minimizing the following cost function:

8 $$mi{n}_{E,D}||R-D\sigma (E\,(R{))||}_{O}^{2}+\frac{\lambda }{2}(||E{||}_{F}^{2}+||D{||}_{F}^{2})$$

where, *E*, *D* and *λ* have the same usual meaning (refer to the previous section), ||·||_*O*_ implies that loss is calculated only for the non-zero counts present in the sparse expression matrix *M*$\circ$*X* and *σ* is the sigmoid activation function applied at the encoder layer, in the neural network.

To prevent overfitting on the non-zero values present in the count matrix, we regularize the learned encoder and decoder matrices.

Once the Encoder and Decoder matrices are learned after training, the imputed expression matrix is given by equation 9. This $\tilde{X}$ consists of imputed or predicted count values at all positions.

9 $$\tilde{X}=D\sigma (E(R))$$

Figure 1 shows the AutoImpute pipeline. The input raw gene expression matrix is filtered for bad genes, normalized by library size, pruned by gene-selection, and log transformed. This processed matrix is then fed into the AutoImpute model (an over-complete autoencoder with the aforementioned 8 cost function) to give the imputed gene expression matrix. Here, the number of input and output nodes have been set to the number of genes in the processed data (1000 genes with the highest dispersion).

Although there is no theoretical insight as to why autoencoders should perform better than matrix completion or matrix factorization, we have an explanation. Assume that the expression matrix has a significantly larger number of cells than the number of genes, i.e., $m\ll n$. The number of variables that needs to be estimated by matrix factorization is *m*.*r* (for *U*) + *n*.*r* (for *V*). Nuclear norm minimization, being largely based on the same concept requires estimation of almost the same number of parameters. The number of independent variables that need to be estimated for the autoencoder is simply 2 × *m*.*r* which is much smaller than the number of variables in matrix factorization or nuclear norm minimization. In a data restricted scenario such as ours, where the model is prone to over-fitting, fewer parameters mean that it will be less susceptible and hence would generalize better leading to better performance.

### Training and Hyper-parameter Selection

The autoencoder network consists of a fully-connected multi-layer perceptron (MLP), with three layers: input, hidden and the output layer. It is trained using gradient descent with gradients computed by back-propagation to reach the minimum of the cost function (equation 8). RMSProp Optimizer was used to adjust the learning rate, such that, we avoid getting stuck at local minima and reach the minimum of the cost function faster. Both *E* - encoder matrix and *D* - decoder matrix were initialized from a random normal distribution.

The hyper-parameter selection was done after doing an extensive grid search on the following hyper-parameters:

- *λ* - the regularization coefficient, to control the contribution of the regularization term in the loss or cost function.
- Size of the hidden layer or latent space dimensionality.
- Initial value of learning rate.
- Threshold value - We stop the gradient descent after the change in loss function value in consecutive iterations is less than the threshold value, implying convergence.

The best results were observed on the hyper-parameter choices shown in Table 2.

**Table 2:** Hyper-parameter choices for different datasets.

Datasets	*λ*	Size of hidden layer	Initial learning rate	Threshold
Blakeley	1	2000	10^−4^	10^−4^
Jurkat-293T	1000	4000	10^−3^	10^−3^
Kolodziejczyk	0	8000	10^−4^	10^−4^
PBMC	12000	3000	10^−5^	10^−3^
Preimplantation	2000	4000	10^−3^	10^−3^
Quake	2000	1500	10^−4^	10^−4^
Usoskin	2000	3000	10^−4^	10^−4^
Zeisel	1000	1500	10^−4^	10^−4^

### Dataset description

The aforementioned experiments were performed on nine different single-cell RNA sequencing datasets, which are described in detail below:

1. **Blakeley:** Single-cell RNA sequencing was performed on a human embryo to define three cell lineages of the human blastocyst^36^: pluripotent epiblast (EPI) cells that form the embryo proper, and extraembryonic trophectoderm (TE) cells and primitive endoderm (PE) cells that contribute to the placenta and yolk sac, respectively. This data with 30 cells, was shared by the authors of^13^.
2. **Jurkat-293T data:** This dataset contains expression profiles of Jurkat and 293T cells, mixed *in vitro* in equal proportions. This data contains 3,388 cells annotated according to the expressions of cell-type specific markers^37^ - the cells expressing CD3D are assigned Jurkat, while those expressing XIST are assigned 293T.
3. **Kolodziejczyk:** Single-cell RNA sequencing of mouse Embryonic Stem Cells (mESCs)^38^ forms the contents of this dataset. These mESCs were cultured under three different conditions: serum/LIF, 2i, and the alternative ground state a2i. Despite sharing a common origin and defining properties, mESCs propagated under different culture conditions also differ, and these culture conditions serve as cell annotations for this dataset.
4. **PBMC data:** This single-cell RNA sequencing data features approximately 68,000 PBMCs (peripheral blood mononuclear cells), freshly collected from a healthy donor. Single-cell expression profiles of 11 purified subpopulations of PBMCs were used as a reference for cell type annotation^37^.
  The dataset is available at 10x Genomics website.
5. **Preimplantation data:** This is a single-cell RNA sequencing data of mouse preimplantation embryos. It contains expression profiles of around 300 cells from zygote at nine stages: early 2-cell stage, middle 2-cell stage, late 2-cell stage, 4-cell stage, 8-cell stage, 16-cell stage, early blastocyst, middle blastocyst and late blastocyst stages. The first generation of mouse strain crosses were used for studying monoallelic expression.
  The count data is available at the Gene Expression Omnibus (GSE45719)^39^.
6. **Quake:** Single-cell RNA sequencing on healthy human brain cell samples was done^40^ to examine the heterogeneity of the adult human cortex. Healthy adult temporal lobe tissue was obtained from epileptic patients during temporal lobectomy for medically refractory seizures, and their cells were classified into all of the major neuronal, glial, and vascular cell types in the brain. This data is available at Gene Expression Omnibus under the accession number GSE67835.
7. **Trapnell**: This single-cell RNA sequencing data is of primary human myoblasts^19^. The cells obtained from culturing differentiating myoblasts were dissociated and individually captured at 24-hour intervals four times. At each of these four times, 50–100 cells were captured following the serum switch using the Fluidigm C1 microfluidic system.
  This data is available at Gene Expression Omnibus under the accession number GSE52529.
8. **Usoskin:** This data of mouse neurons^41^ was obtained by performing RNA sequencing on 799 dissociated single-cells dissected from the mouse lumbar dorsal root ganglion (DRG) distributed over a total of nine 96-well plates. The cell labels (clusters of mouse lumbar DRG-NF, NP, TH, PEP populations) were computationally derived and assigned by performing PCA classification on single mouse neurons.
  RPM normalized counts with full sample annotation are available at http://linnarssonlab.org/drg/ (also available under the accession number GSE59739).
9. **Zeisel:** Quantitative single-cell RNA sequencing has been used to classify cells in the mouse somatosensory cortex (S1) and hippocampal CA1 region based on 3005 single-cell transcriptomes^42^. Individual RNA molecules were counted using unique molecular identifiers (UMIs) and confirmed by single-molecule RNA fluorescence *in situ* hybridization (FISH). A divisive biclustering method based on sorting points into neighborhoods (SPIN) was used to discover molecularly distinct, nine major classes of cells.

Raw data is available under the accession number GSE60361.

### Data preprocessing

We perform the following steps for preprocessing of raw scRNA-seq data.

- **Gene filtering:** If a gene is detected with ≥3 reads in at least three cells, we consider it expressed, and ignore all the un-expressed genes.
- **Median Normalization:** Expression matrices are normalized by first dividing each read count by the total counts in each cell, and then by multiplying with the median of the total read counts across cells.
- **Gene Selection:** For each expression data top 1000 high-dispersion (coefficient of variance) genes are kept^43^ for imputation and further analyses.
- **Log Normalization:** A copy of the matrices, were log_2_ transformed following the addition of 1 as pseudo count.
- **Imputation:** For various experiments, log-transformed expression matrix was used as input for imputation by one of the methods.

## Software

The source code of AutoImpute is available at the GitHub repository: https://github.com/divyanshu-talwar/AutoImpute.

## Future Work

The current version of our AutoImpute model has three layers of nodes, and hence essentially follows a shallow neural network architecture. Motivated by tremendous success of deep learning in image and speech recognition^44–46^ and stacked autoencoders in the filed of collaborative filtering^28,47^ (for matrix completion), we propose to deploy a deeper version of our model for imputing missing values in the future work. Such deeper model is expected to improve the results even further.

## Electronic supplementary material

Supplementary Material

## Data Availability

All datasets have been downloaded from 10x Genomics website and the Gene Expression Omnibus as mentioned in the Dataset description subsection.
