# Supplementary material for: AutoImpute: Autoencoder based imputation of single-cell RNA-seq data
Source: Sci Rep. 2018 Nov 5;8:16329. doi: 10.1038/s41598-018-34688-x (PMC6218547; doi:10.1038/s41598-018-34688-x)
Supplement: Supplementary file 1 — Supplementary Material [file 41598_2018_34688_MOESM1_ESM.pdf]

AutoImpute: Autoencoder based imputation of single-cell RNA-seq data

Supplementary Material

Divyanshu Talwar[1,†], Aanchal Mongia[1,†], Debarka Sengupta[1,3 *], and Angshul Majumdar[2]

{divyanshu15028, aanchalm, debarka, angshul}@iiitd.ac.in

[1] Department of Computer Science and Engineering, Indraprastha Institute of Information Technology, Delhi, India.
[2] Department of Electronics and Communications Engineering, Indraprastha Institute of Information Technology, Delhi, India.
[3] Center for Computational Biology, Indraprastha Institute of Information Technology, Delhi, India.
[†] These authors contributed equally to this work.
[*] Corresponding Author

Supplementary Table S 1: **Separation of "true zeros" from dropouts** - Fraction of zeros (values between 0 and 0.5) in single cell expression matrix against the median bulk expression. The genes are divided into 10 bins based on median bulk genes expression (first bin has only 0 expression gene).

**Median Bulk Expression Bins**	**Algorithms**				
	**No imputation**	**AutoImpute**	**scImpute**	**Magic**	**drImpute**
**0**	0.9471	0.9471	0.9194	1.0000	0.8585
**0 - 1**	0.9589	0.9378	0.8945	1.0000	0.8302
**1 - 2**	0.9298	0.9350	0.8843	0.9545	0.8343
**2 - 3**	0.9753	0.9777	0.9537	1.0000	0.9274
**3 - 4**	0.9507	0.9439	0.8890	1.0000	0.7778
**4 - 6**	0.9301	0.8746	0.7644	0.9924	0.6247
**6 - 8**	0.8253	0.5726	0.4807	0.8941	0.3253
**8 - 10**	0.6086	0.2145	0.1665	0.5344	0.0645
**10 - 12**	0.3675	0.1060	0.0775	0.1746	0.0120
**12 - max**	0.1456	0.0480	0.0329	0.0234	0.0024

Supplementary Table S 2: **Gene expression recovery** - Comparison of NMSE, RMSE and MAE calculated between the full unmasked single cell RNA-seq data and the recovered expression matrices with masked matrices as inputs (with varying masking percentage), at the masked locations, for AutoImpute, scImpute, and Magic algorithms.

Masking %	NMSE			RMSE			MAE		
	AutoImpute	scImpute	Magic	AutoImpute	scImpute	Magic	AutoImpute	scImpute	Magic
**Blakeley**									
10	**1.80** ± **0.1270**	1.4833	0.6173	**15.34** ± **0.8754**	12.3598	1.26E+03	**12.21** ± **0.8522**	9.7188	497.2412
20	**1.82** ± **0.2114**	2.0525	0.5952	**15.55** ± **1.7696**	17.4443	1.42E+03	**12.35** ± **1.387**	11.7828	520.2138
30	**1.84** ± **0.1226**	2.8635	0.6305	**15.34** ± **1.0574**	24.3536	1.42E+03	**12.37** ± **0.6635**	13.1893	530.4287
40	**1.99** ± **0.0651**	3.5833	0.6771	**16.97** ± **0.4960**	30.6860	1.52E+03	**13.55** ± **0.3941**	13.1764	539.3752
50	**2.22** ± **0.0350**	12.4788	0.7338	**18.86** ± **0.3273**	105.8491	1.73E+03	**15.01** ± **0.2601**	24.1704	575.0085
**Jurkat-293T**									
10	**0.39** ± **0.0143**	2.0607	0.5239	**1.09** ± **0.0402**	3.5464	3.2438	**0.96** ± **0.0382**	0.3870	0.4744
20	**0.40** ± **0.0377**	5.6608	0.5825	**1.19** ± **0.1063**	9.1853	3.4002	**1.04** ± **0.0508**	0.8851	0.4848
30	**0.44** ± **0.0895**	13.6579	0.6332	**1.25** ± **0.0500**	20.7366	3.4586	**1.34** ± **0.2031**	1.7311	0.4855
40	**0.48** ± **0.5047**	54.4675	0.6791	**1.41** ± **0.4410**	76.5812	3.4294	**1.12** ± **0.6816**	3.9182	0.4728
50	**0.49** ± **0.6816**	449.9235	0.7199	**1.63** ± **0.6029**	577.3294	3.3262	**1.79** ± **0.9975**	10.9636	0.4441
**Kolodziejczyk**									
10	**2.82** ± **0.0127**	1.6747	0.3711	**25.13** ± **0.1063**	14.9218	1.23E+03	**20.04** ± **0.0916**	9.4494	192.2411
20	**3.14** ± **0.0221**	3.0888	0.4597	**27.92** ± **0.2057**	27.4045	1.58E+03	**22.24** ± **0.1613**	13.011	203.654
30	**3.39** ± **0.0177**	6.1239	0.5940	**30.21** ± **0.1418**	54.4813	1.86E+03	**24.09** ± **0.1347**	19.0343	221.3524
40	**3.62** ± **0.0020**	13.4705	0.6770	**32.22** ± **0.0300**	119.9824	2.20E+03	**25.66** ± **0.0297**	29.8625	238.5226
50	**3.87** ± **0.0043**	90.4541	0.7238	**34.40** ± **0.0502**	803.8800	2.23E+03	**27.43** ± **0.0500**	69.1118	257.9593
**Quake**									
10	**0.95** ± **0.0007**	24.82263737	0.7731	**8.51** ± **0.0705**	2.23E+02	8.38E+03	**6.78** ± **0.0268**	63.44775511	2013.0823
20	**0.99** ± **0.0017**	1.29E+02	0.8031	**8.89** ± **0.0238**	1.15E+03	8.14E+03	**7.08** ± **0.0195**	1.25E+02	1978.092744
30	**1.02** ± **0.0013**	6.06E+02	0.8426	**9.18** ± **0.0144**	5.43E+03	8.52E+03	**7.30** ± **0.0159**	3.63E+02	1998.363221
40	**1.07** ± **0.0008**	3.13E+03	0.8865	**9.56** ± **0.0157**	2.80E+04	9.46E+03	**7.60** ± **0.0044**	1.24E+03	2011.990659
50	**1.10** ± **0.0015**	2.18E+06	0.9075	**9.85** ± **0.0013**	1.95E+07	9.68E+03	**7.83** ± **0.0049**	1.35E+05	2026.776616
**Preimplantation**									
10	**0.29** ± **0.0094**	1.6424	0.777	**2.42** ± **0.0888**	6.8395	1.71E+03	**1.90** ± **0.9902**	0.7164	286.8199
20	**0.29** ± **0.0057**	6.6387	0.8052	**2.42** ± **0.0451**	26.0432	1.85E+03	**1.85** ± **0.0661**	2.0347	313.6260
30	**0.31** ± **0.1280**	54.469	0.8277	**2.57** ± **0.1050**	200.0148	1.96E+03	**1.91** ± **0.1550**	6.2662	330.6937
40	**0.31** ± **0.0180**	120.2089	0.8501	**2.58** ± **0.1530**	408.693	2.00E+03	**2.04** ± **0.1256**	13.8265	341.3199
50	**0.31** ± **0.014**	1.21E+04	0.8672	**2.60** ± **0.1200**	3.77E+04	2.08E+03	**2.24** ± **0.1341**	6.2662	344.7977
**Usoskin**									
10	**2.23** ± **0.0182**	41.22689	0.6443	**9.69** ± **0.0564**	1.60E+02	52.5217	**7.69** ± **0.0256**	24.81428717	14.1806
20	**2.40** ± **0.0022**	3.18E+03	0.6720	**10.40** ± **0.0411**	1.30E+04	55.2395	**8.26** ± **0.0310**	1.83E+02	14.7904
30	**2.53** ± **0.0121**	3.96E+07	0.7645	**10.95** ± **0.0325**	1.67E+08	68.8496	**8.72** ± **0.0293**	8.18E+05	15.2874
40	**2.63** ± **0.0119**	8.06E+05	0.7696	**11.37** ± **0.0439**	3.42E+06	66.2696	**9.05** ± **0.0444**	1.56E+04	15.8968
50	**2.75** ± **0.0764**	2.56E+06	0.8590	**11.93** ± **0.0586**	1.09E+07	84.2230	**9.50** ± **0.0514**	5.70E+04	17.0062
**Zeisel**									
10	**1.42** ± **0.0077**	19.3472739	0.9038	**4.23** ± **0.0238**	57.51650418	1.68E+01	**3.07** ± **0.0326**	13.02629774	1.9248
20	**1.77** ± **0.0028**	51.45857788	0.926	**5.25** ± **0.0162**	152.4683696	1.72E+01	**4.05** ± **0.0134**	23.33065176	2.0222
30	**1.99** ± **0.0095**	1025.814158	0.9409	**5.91** ± **0.0377**	3043.370528	1.79E+01	**4.63** ± **0.0320**	76.24520225	2.1272
40	**2.20** ± **0.0008**	12127.06557	0.9554	**6.52** ± **0.0024**	35970.72819	1.81E+01	**5.14** ± **0.0023**	324.6180099	2.2441
50	**2.34** ± **0.0235**	48999902.91	0.9612	**6.96** ± **0.0670**	1.45E+08	1.73E+01	**5.49** ± **0.0553**	288527.3319	2.364

Supplementary Table S 3: **Median of coefficient of variation** across cells from a random subpopulation selected from each dataset imputed using the following algorithms.

Datasets	No Imputation	scImpute	drImpute	Magic	AutoImpute
Blakeley	3.3114	2.1918	2.4142	3.35E-06	2.7507
Jurkat-293T	2.8399	12.0685	1.9193	0.031	0.0164
Kolodziejczyk	78.0416	14.5777	10.127	0.2526	4.6243
PBMC	58.3697	–	–	–	0.0011
Preimplantation	6.6962	5.0814	4.1446	0.0595	0.0012
Quake	11.5473	6.4453	4.0275	0.0106	8.8568
Usoskin	28.9626	14.4071	27.7187	0.2375	10.9204
Zeisel	32.4045	4.891	1.8093	0.0728	11.0032

Supplementary Table S 4: **Average silhouette index values** showing cell-type separability on cell groupings corresponding to first two principal components of expressions.

Datasets	No Imputation	scImpute	drImpute	Magic	AutoImpute
Blakeley	0.93	0.67	0.44	0.20	0.87
Jurkat-293T	0.94	0.93	0.70	0.90	0.96
Kolodziejczyk	0.24	0.46	0.43	-0.18	0.37
Preimplantation	-0.007	-0.06	0.03	0.01	0.08
Quake	-0.26	0.36	0.32	-0.03	0.33
Usoskin	0.33	0.12	-0.13	-0.08	0.36
Zeisel	-0.14	0.19	0.08	-0.16	0.34
